# Documenting decapod biodiversity in the Caribbean from DNA barcodes generated during field training in taxonomy

**DOI:** 10.3897/BDJ.8.e47333

**Published:** 2020-01-07

**Authors:** Dagoberto E. Venera-Pontón, Amy C. Driskell, Sammy De Grave, Darryl L. Felder, Justin A. Scioli, Rachel Collin

**Affiliations:** 1 Smithsonian Tropical Research Institute, Balboa, Panama Smithsonian Tropical Research Institute Balboa Panama; 2 University of Louisiana at Lafayette, Lafayette, United States of America University of Louisiana at Lafayette Lafayette United States of America; 3 Laboratories of Analytical Biology, Department of Invertebrate Zoology, Smithsonian Institution, Washington, D.C., United States of America Laboratories of Analytical Biology, Department of Invertebrate Zoology, Smithsonian Institution Washington, D.C. United States of America; 4 Oxford University Museum of Natural History, Oxford, United Kingdom Oxford University Museum of Natural History Oxford United Kingdom

**Keywords:** cytochrome c oxidase I, Panama, Bocas del Toro, shrimps, crabs

## Abstract

DNA barcoding is a useful tool to identify the components of mixed or bulk samples, as well as to determine individuals that lack morphologically diagnostic features. However, the reference database of DNA barcode sequences is particularly sparsely populated for marine invertebrates and for tropical taxa. We used samples collected as part of two field courses, focused on graduate training in taxonomy and systematics, to generate DNA sequences of the barcode fragments of *cytochrome c oxidase* subunit I (COI) and mitochondrial ribosomal 16S genes for 447 individuals, representing at least 129 morphospecies of decapod crustaceans. COI sequences for 36% (51/140) of the species and 16S sequences for 26% (37/140) of the species were new to GenBank. Automatic Barcode Gap Discovery identified 140 operational taxonomic units (OTUs) which largely coincided with the morphospecies delimitations. Barcode identifications (i.e. matches to identified sequences) were especially useful for OTUs within *Synalpheus*, a group that is notoriously difficult to identify and rife with cryptic species, a number of which we could not identify to species, based on morphology. Non-concordance between morphospecies and barcode OTUs also occurred in a few cases of suspected cryptic species. As mitochondrial pseudogenes are particularly common in decapods, we investigate the potential for this dataset to include pseudogenes and discuss the utility of these sequences as species identifiers (i.e. barcodes). These results demonstrate that material collected and identified during training activities can provide useful incidental barcode reference samples for under-studied taxa.

## Introduction

A shortage of taxonomic expertise is one of the current challenges facing those engaged in identifying, classifying, utilising and conserving the world’s biodiversity (Giangrande 2003, Vernooy et al. 2010, Cardoso et al. 2011, Ebach et al. 2011, Wägele et al. 2011, Sluys 2013). The recent application of DNA techniques, the compilation of large taxonomic databases (Costello et al. 2013) and the use of bioinformatics approaches like GIS, have rejuvenated interest in taxonomic data. Unfortunately, this increase in relevance and interest has been counteracted by the gradual loss of integrative taxonomic expertise (Drew 2011, Coleman 2015). This recent decline has limited attempts to document the world's biodiversity and limits the rate at which high-profile initiatives, such as the Census for Marine Life, Barcode of Life (BOLD; Ratnasingham and Hebert 2007) and WoRMS, can generate, agglomerate and synthesise biodiversity knowledge. There is a particular need for new experts specialising in marine biodiversity, where it is estimated that 30-60% (Appeltans et al. 2012) or even 90% (Mora et al. 2011) of eukaryotic species remain to be described or discovered. There is also a particular shortage of taxonomists and data from developing countries and countries with economies in transition.

DNA barcoding is a useful tool for the identification of samples that cannot be identified, based on traditional morphological methods (Bucklin et al. 2011, Bucklin et al. 2016, Geiger et al. 2016). Short, easily amplifiable fragments that vary amongst closely related species are sequenced from specimens identified by experts and then used as a reference set to compare with sequences from unidentified or unidentifiable samples (Hebert and Gregory 2005, Hajibabaei et al. 2007, Bucklin et al. 2011, Bucklin et al. 2016, Geiger et al. 2016). For animals, the most widely used barcode is *cytochrome c oxidase* subunit I (COI; Hebert et al. 2003), followed by the 16S large subunit ribosomal RNA (16S), another mitochondrial marker (see Mantelatto et al. 2017, Collin et al. 2018, Collin et al. 2019, Morín et al. 2019). This approach is useful in a variety of contexts, including identifying components of gut contents and bulk environmental samples. However, the global DNA barcode database (BOLD; Ratnasingham and Hebert 2007) is still sparsely populated, specifically for invertebrates and especially for tropical taxa. In many cases, even common, relatively easily identified and well-known taxa do not yet have sequences of the DNA barcode fragment of COI publicly available in GenBank. For example, Raupach and Radulovici (2015) showed a particular lack of DNA barcode studies for crustaceans from the Caribbean, amongst other regions. Therefore, activities that can aid in generating DNA reference barcodes for commonly encountered species, even without a comprehensive effort to exhaustively document species in a particular group or fauna, can make a significant contribution to the utility of the barcode database. In addition, improved taxonomic coverage may assist in narrowing down the possible identities of unknown samples that do not match any reference sample.

Here, we used material collected as part of graduate taxonomy training workshops in Bocas del Toro, Panama to generate a reference set of DNA barcodes of common shallow-water decapods of the Caribbean coast of Panama. Decapods are one invertebrate group that, despite its importance and high diversity, still has low DNA barcode coverage in the tropics (Raupach and Radulovici 2015). Our hope was that by combining the two, not only could trainees become familiar with processing material for subsequent DNA extraction, but that the contribution of biodiversity data to global databases would help garner continued support and help make training activities more sustainable (Cancian de Araujo et al. 2018). Due to the wide geographic ranges of many taxa throughout the Caribbean and the potential for gene flow between Bocas del Toro and other parts of the region via dispersal of planktonic larvae on ocean currents (Cowen et al. 2007, Cowen and Sponaugle 2009, Schill et al. 2015), our barcode library may be useful in other zones of the Caribbean Sea.

## Material and methods

### Collection

Specimens for DNA barcoding were collected during two workshops of the *Training in Tropical Taxonomy* programme run by the Smithsonian Tropical Research Institute in Bocas del Toro, Panama. The “*Shrimp Taxonomy (Caridea, Dendrobranchiata and Stenopodidea)*” course in 2008 included 13 students from eight countries (Mexico, UK, US, Colombia, Slovenia, Brazil, Australia and Costa Rica) and the “*Taxonomy and Biology of Decapod Crustaceans*” course included 13 students from five countries (US, Colombia, Brazil, Argentina and Costa Rica) in 2011. These 2-week workshops, each led by one of us (SDG and DF, respectively) and co-instructed by A. Anker and F. Mantelatto, respectively, were aimed at graduate student training but also included undergraduate students and post-doctoral professionals seeking training in systematics and identification of the focal groups. During two weeks, students collected and identified specimens as part of their training and, when the animals were intact and well-enough preserved to make useful vouchers, tissue samples were taken by us for DNA barcoding (see section on DNA sequencing). Therefore, unlike other studies that included training sessions in DNA barcoding (e.g. Harris and Bellino 2013), our study simply made use of specimens collected and identified under the supervision of taxonomic experts during taxonomy training workshops. All specimens were collected from Bahia Almirante, especially from sites in and around Isla Colon and Isla Bastimentos. No documentation of sampling effort was made, as collections were opportunistic and arranged around the other activities of the courses. The marine invertebrate diversity of this area has been documented in some detail (e.g. Collin et al. 2005, Rocha et al. 2005, Bonnet and Rocha 2011, Goodheart et al. 2016). A detailed checklist is available for shrimps (De Grave and Anker 2017), but no checklists or comprehensive surveys are available for brachyurans or anomurans.

Vouchers from the decapod course are deposited in the decapod collection of the University of Louisiana at Lafayette (ULL) which is currently being transferred to the Smithsonian Natural History Museum (USNM). Reference numbers from the UL collection are provided in the dataset associated to this research (dx.doi.org/10.5883/DS-CRUSTACE and Table 1). Many vouchers from the shrimp course are currently stored in the Zoological Collection of the Oxford University Museum of Natural History (OUMNH.ZC see Table 1). However, a number of shrimp samples were transferred to the Museu Nacional de Brazil and were subsequently lost in the fire that destroyed the museum in 2018. This lost material is listed without voucher numbers (Table 1). Additionally, a few shrimp samples are stored in the Natural History Museum of Vienna (NHMW).

### DNA Sequencing

Specimens from the shrimp course were extracted in Panama using a Biosprint 96 and a DNA Blood Kit (Qiagen) and the DNA extracts were shipped to the Smithsonian’s Laboratories of Analytical Biology (LAB) for PCR and sequencing. For the decapod course, small pieces of tissue were preserved in 150 μl of M2 extraction buffer (AutoGen), stored frozen and shipped to LAB for extraction and sequencing. Samples were extracted using an AutoGenprep 965 extraction robot after overnight digestion in AutoGen buffer with proteinase-K. We sequenced two gene fragments. The DNA barcode fragment of the *cytochrome c oxidase* subunit I (COI) was amplified using primarily the primer pair jgLCO1490/jgHCO2198 (Geller et al. 2013), although the pair dgLCO1490/dgHCO2198 (Meyer et al. 2005) was also used. The 10 μl PCR mix included 1 μl Biolase Taq (Promega), 0.1 μl BSA and 0.3 μl of each 10 mM primer. For amplification and sequencing of 16S, the primer pair 16S AR/16S BR (Palumbi et al. 1991) was used. The mix for 16S was the same as for COI with the addition of 0.5 µl 50 mM MgCl_2_. The annealing temperature for nearly all reactions for all three gene regions was 50°C, although occasionally it was raised to 52°C when it appeared that co-amplification was occurring. Sequencing followed the methods described in Collin et al. (2018), Collin et al. (2019), and Morín et al. (2019).

### Analysis

Sequences were screened for quality and contigs of forward and reverse sequences were produced using Sequencher 5.4.6 (Gene Codes). Only sequences with a length of more than 90% of the expected length and with a Phred quality score of at least 30 for more than 85% of the bases were combined into contigs and used for analyses. To check for potential contamination, sequences were compared within the BOLD workbench (www.boldsystems.org; Ratnasingham and Hebert 2007) to all taxa sequenced in our project; likewise, sequences were compared to publicly available sequences using BLASTn searches in GenBank. The few sequences with > 95% identity to non-decapods were eliminated from subsequent analyses. COI sequences were also checked with the methods of Song et al. (2008) to determine whether they displayed detectable pseudogene traits (Buhay 2009).

As DNA barcoding is usually a distance-based approach, we constructed a neighbour-joining tree (BIONJ, Gascuel 1997) with Jukes-Cantor distances to preliminarily recognise distinct OTUs. Neighbour-joining trees with Kimura's-two-parameters distances were also constructed and produced the same results as the Jukes-Cantor distances. The tree nodes were further verified with non-parametric bootstrapping, using the Felsenstein's method (Felsenstein 1985, Efron et al. 1996). COI alignments were inferred with the BOLD aligner [amino acid-based Hidden Markov Model (Ratnasingham and Hebert 2007)], whereas 16S alignments used the Kalign algorithm (Lassmann and Sonnhammer 2005) with the default settings of the BOLD workbench. Alignments were subsequently corrected manually. Operational Taxonomic Units (OTUs) were delimited with the Automatic Barcode Gap Discovery method (Puillandre et al. 2011) using the following parameters: P_min_ = 0.001; P_max_ = 0.1 for COI and 0.05 for 16S; X = 1.125 for COI and 1.5 for 16S; Steps = 10. P_min_ and P_max_ were chosen with the help of a histogram of distances and X was smaller in COI because the default 1.5 value did not provide enough sensitivity to partition the data (see Puillandre et al. 2011).

Whenever an OTU differed between COI and 16S, the OTU was accepted only if it diverged from every other sequence by at least 0.05 substitutions per site in COI or 0.03 in 16S. If the discrepancy remained unresolved, then we accepted the option producing fewer OTUs. The final consensus OTUs were compared to the system of Barcode Index Numbers (BINs) assigned in BOLD (Ratnasingham and Hebert 2013) and to our morphological identifications, in order to detect potentially cryptic species or previously unrecognised diversity.

## Data resources

The DNA sequences associated with this paper are deposited in the Barcode of Life Database (dataset dx.doi.org/10.5883/DS-CRUSTACE) (Ratnasingham and Hebert 2007, Ratnasingham and Hebert 2013) and GenBank (www.ncbi.nlm.nih.gov/genbank) (accession numbers MN183805-MN184218 for COI and MK971234-MK971659 for 16S).

## Results and Discussion

**Figure 1.**
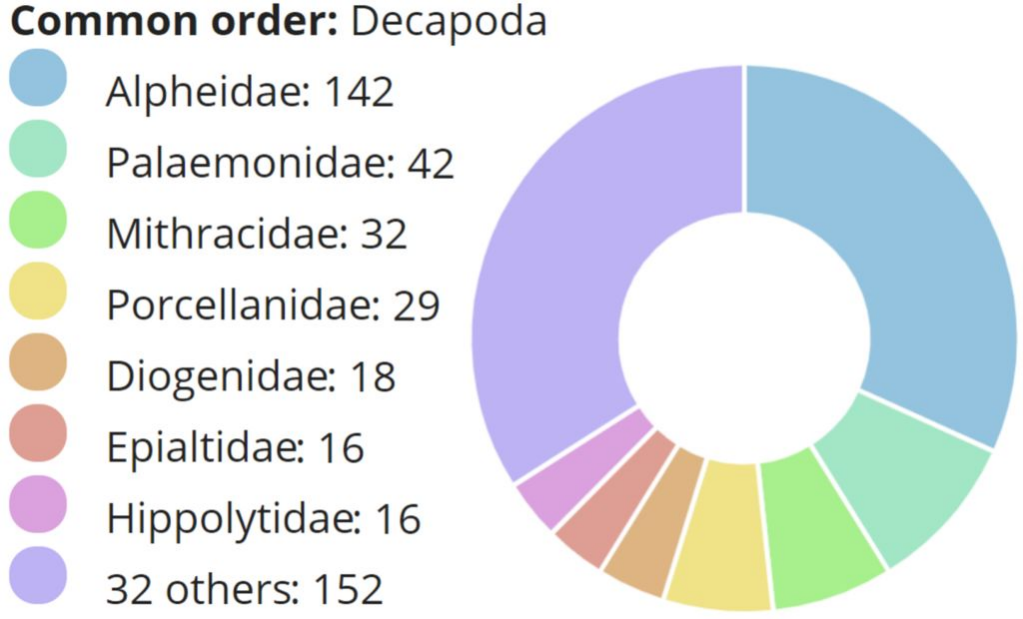
Pie chart indicating the number of individuals present on this study for each decapod family.

**Figure 2.**
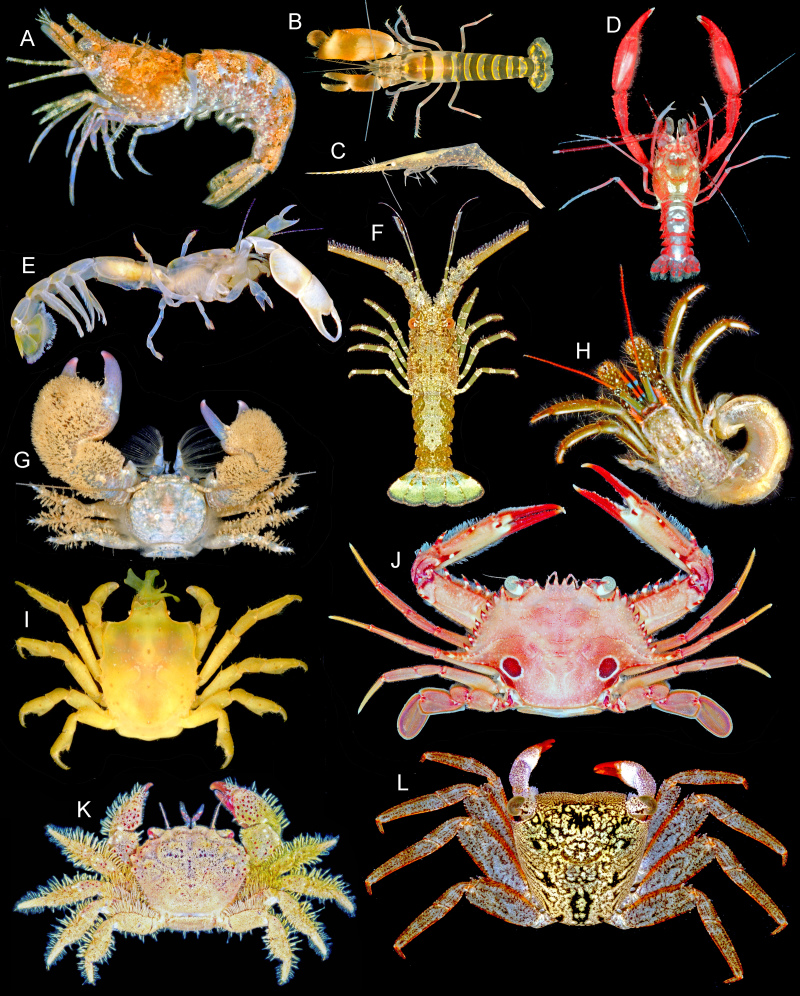
Representative decapod crustacean specimens from Bocas del Toro, Panama. **A.** *Sicyonia laevigata*, ULLZ13484; **B.** *Alpheus thomasi*, ULLZ18292; **C.** *Tozeuma carolinense*, ULLZ18291; **D.** *Microprosthema semilaeve*, ULLZ10770; **E.** *Pseudobiffarius caesari*, ULLZ13480; **F.** *Panulirus argus* juvenile, ULLZ13319; **G.** *Pachycheles tuerkayi*, ULLZ6098; **H.** *Clibanarius antillensis*, ULLZ16971; **I.** *Acanthonyx petiverii*, ULLZ12015; **J.** *Achelous sebae*, ULLZ17128; **K.** *Pilumnus holosericus*, ULLZ17614; **L.** *Aratus pisonii*, ULLZ14799.

A total of 447 individuals, morphologically identified to 129 species, were successfully sequenced for at least one marker, including 47 species of shrimps, 57 brachyuran crabs, one achelate lobster, four axiid mudshrimp, one gebiid mudshrimp and 19 anomuran crabs (Table 1, Figs 1, 2). Shrimps included the infra-orders Caridea, Dendrobranchiata and Stenopodidea. Amongst successfully sequenced individuals, 99 were identified to genus, but could not be confidently assigned to a species based on morphology. The Automatic Barcode Gap Discovery method delimited 141 OTUs with COI and 140 OTUs with 16S; likewise, our COI sequences were assigned to 146 Barcode Index Numbers (BINs) in BOLD. The larger number of OTUs and BINs suggest there are ~10 potentially cryptic species or species with unusually high levels of genetic diversity in this dataset.

Eighty seven of our consensus OTUs matched COI sequences already in GenBank with an identity of > 95% (see Table 1). Of these, our identification and the name on the GenBank sequence were concordant for 77 of the OTUs, including seven cases in which our identification provided better taxonomic resolution than the GenBank sequence. In many cases, these represent samples of the same taxa from other Caribbean regions confirming the conspecific status of animals from different parts of the same biogeographic region. In ten cases where our identification was not concordant with the name of a COI GenBank sequence >95% identical, the discrepancy typically occurred at the species level while the higher taxonomic ranks remained concordant. Two OTUs did not have sequences in COI: one was a singleton identified as *Leander paulensis* and the other included two specimens identified as *Pilumnus reticulatus* and *P. pannosus*. The remaining 51 OTUs for which we have COI sequences were < 95% identical to another sequence in GenBank and therefore considered to be new additions.

The results for the 16S analysis were relatively similar: 99 consensus OTUs were > 97% identical to 16S sequences available in GenBank and for 89 of them, the morphological identification coincided with the name of the GenBank sequence, whereas the other ten OTUs showed discrepancies at the species level with the GenBank sequence, but remained concordant at higher taxonomic ranks. Four singleton OTUs, identified as *Stenopus scutellatus*, *Inachoides* sp., *Austinixa aidae* and *Speleophorus nodosus* did not have sequences in 16S. The remaining 16S sequences (belonging to 37 OTUs) were > 97% similar to other sequences in GenBank; thus these were considered new additions.

Our dataset contributed 56 new BINs to BOLD and provided 38 new species for at least one marker in GenBank (Table 1). One hundred and thirty seven of our 140 OTUs are associated with only one morphospecies name. This coincides with our visual observations of the COI and 16S neighbour-joining trees, which showed that our morphospecies identifications are largely concordant with clusters of very similar sequences. These clusters differed from other such clusters by ~0.10 substitutions per site in COI and ~0.05 in 16S (Fig. 3). Such concordance between morphospecies and OTUs, and the magnitude of the observed interspecific divergence are similar to those reported by Costa et al. (2007) and Matzen da Silva et al. (2011). Nevertheless, there are several cases where animals could not be identified to species or in which a single species/species-complex name appeared in different OTUs. These included the following:

For *Synalpheus* spp., 42 individuals fell into eight OTUs. Seven OTUs matched GenBank sequences from in-depth studies of these taxa (Duffy et al. 2000, Morrison et al. 2004, Hultgren et al. 2014, Chak et al. 2017). These were *S. hoetjesi*, *S. paraneptunus*, *S. yano*, S. aff. longicarpus, *S. elizabethae* and S. cf. rathbunae. The final OTU did not match anything in GenBank (identity < 90% in both markers). Our failure to detect more than one additional species in this group suggests that it has been well-sampled in Bocas del Toro.

Specimens of *Alpheus* spp. were split into 18 OTUs. Thirteen OTUs were identified to species, including two OTUs assigned to the same species name (*Alpheus paracrinitus*); one of these OTUs had four individuals, whereas the other was a singleton. *A. paracrinitus* has long been considered an unresolved species complex, including at least four species (Knowlton et al. 1993, Anker 2001). The other five OTUs were identified to genus (*Alpheus* sp.) or as members of a species complex (e.g. *A. packardii complex*). Eleven *Alpheus* OTUs were new for BOLD, adding 11 new BINs to the database. Likewise, eleven *Alpheus* OTUs were < 95% identical to COI or < 97% to 16S sequence in GenBank and, thus, constitute new additions to the database; moreover, our sequences added five new *Alpheus* species names for 16S in GenBank. Only one OTU, identified as *A. paraformosus*, matched a different species name, *A. formosus*, in GenBank (sequence from Leray and Knowlton 2015). Most of the OTUs identified as members of the *A. packardii complex* are < 95% and < 97% identical to COI and 16S sequence in GenBank, respectively. One other OTU, identified as *Alpheus* sp., matched GenBank sequences that were also identified only to genus. Clearly, we are far from having a complete barcode database for this speciose taxon.

The shrimps ***Tozeuma carolinense*** (Fig. 2C) fell into two OTUs, one with eight and the other with one specimen. Both of these OTUs were new for BOLD, adding two new BINs to the database. The 16S sequences for one of these OTUs matched a *T. carolinense* sequence in GenBank with > 99% identity. However, both of our *T. carolinense* OTUs were distinct from the available COI sequences in GenBank with > 95% identity (Fig. 3, Table 1), suggesting that this morphologically distinctive species may include several cryptic species. A similar situation occurred for *Sicyonia laevigata* (Fig. 2A), *Pagurus criniticornis* and *Alpheus paracrinitus*; all these names being assigned to specimens that grouped in two OTUs: one with multiple species and the other being a singleton.

**OTUs with multiple species names**: One OTU included sequences from two specimens, morphologically identified as different species (*Pilumnus dasypodus* and *P. caribaeus*). This OTU matched *P. dasypodus* sequences in GenBank with > 99% identity for both markers. Another OTU identified as *P. caribaeus* in our dataset matched GenBank sequences of that species. One other OTU comprised specimens morphologically identified as 2 species (*P. pannosus* and *P. reticulatus*).

### Pseudogenes in Decapod Barcoding

**Figure 4.**
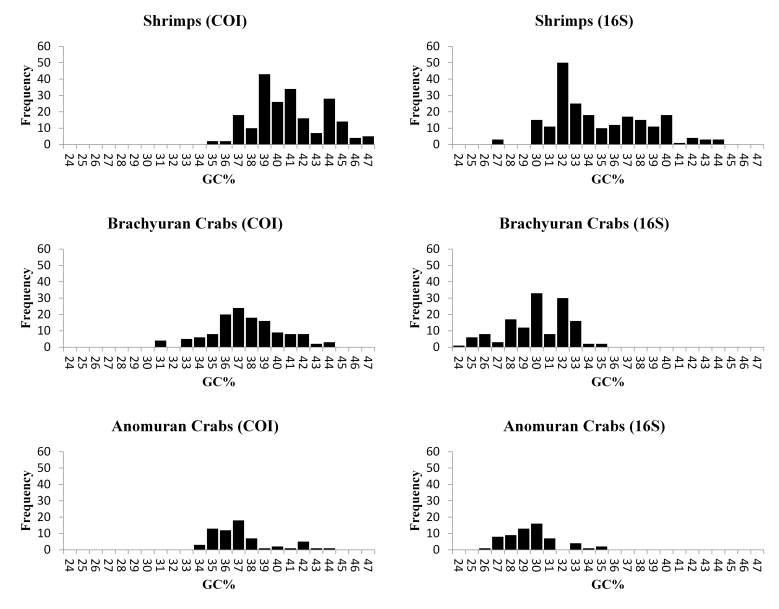
Histogram of the GC content for COI and 16S in three major groups of decapods evaluated in this study. Shrimps included the infra-orders Caridea, Dendrobranchiata and Stenopodidea.

We found no indels in our COI sequences and no stop codons in the corresponding amino acid sequence. Both the COI and 16S sequences showed a range of GC content (GC%) from 23.97-46.48% (Fig. 4), which is similar to the findings of other studies (e.g. Costa et al. 2007, Matzen da Silva et al. 2011). As mitochondrial genes are expected to show a different AT bias than pseudogenes (Bensasson 2001, Song et al. 2008, Matzen da Silva et al. 2011, Liu et al. 2016), those sequences with significantly deviant GC% are potentially more likely to be pseudogenes; nevertheless, a careful examination of all our sequences failed to detect any strong evidence of pseudogenes. The overall concordance between our morphological identifications and the molecular identification of OTUs, based on 16S and COI, further supports the conclusion that pseudogenes were rare or absent in this dataset.

Much has been made of the problem with pseudogenes in decapods (Schneider-Broussard and Neigel 1997, Williams and Knowlton 2001, Song et al. 2008, Schubart 2010, Matzen da Silva et al. 2011, Raupach and Radulovici 2015, but see Schizas 2012). They are undoubtedly more common in decapods than in some other groups of marine invertebrates, where they have seldom been reported. It is also clear that pseudogenes can cause significant problems in phylogenetic reconstructions (Schubart 2010). However, we argue that the problems they pose for DNA barcoding are limited and that the difficulty in determining if a sequence is a pseudogene or not without resorting to cloning, means that barcode datasets for decapods may never be entirely free of pseudogene sequences unless mitochondrial DNA is directly targeted during DNA extraction with a mitochondrial DNA isolation kit.

**Figure 5.**
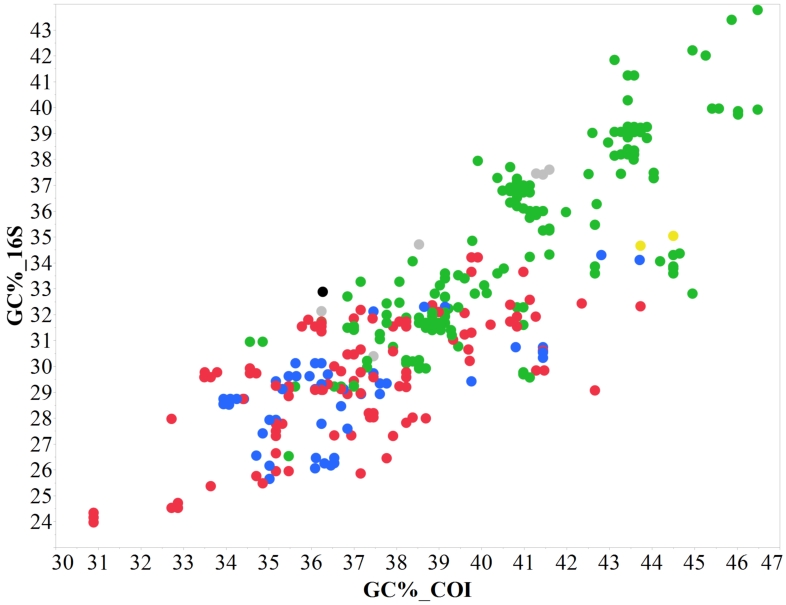
Scatterplot of the GC content (GC%) in COI versus 16S for every individual of this study, successfully sequenced for both markers. In general, the GC% of the two markers appears to be as correlated as expected for two mitochondrial genes and none of the specimens seems to deviate enough to be considered a suspect pseudogene. Major groups are indicated with colours: Brachyura: red, Anomura: blue, Shrimps (Caridea, Dendrobranchiata, and Stenopodidea): green, Axiidea: light-grey, Gebiidae: black, Achelata: yellow.

Song et al. (2008) described a 3-step method for eliminating obvious pseudogenes in protein coding sequences: eliminate sequences with indels and stop codons, eliminate sequences with unusual or highly divergent amino acid sequences and eliminate sequences with unusual GC bias. The first two steps are only applicable to protein coding sequences like COI and cannot be applied to 16S, another commonly used barcoding gene for many groups [e.g. amphibians (Vences et al. 2005), hydrozoans (Zheng et al. 2014) and other marine invertebrates (Brasier et al. 2016)]. The third step is hindered by the difficulty in determining what GC% value is sufficiently different to be considered suspect. The GC% values of our sequences ranged from 24.0-46.5% and their histograms, in general, did not show any distinct gap or dip that could be considered as a clear threshold between coding sequences and pseudogenes. The small gaps and asymmetric distributions observed in some histograms were always associated with interspecific differences in GC%; for example, the large GC% of *Dardanus fucosus*, *Calcinus tibicens* and *Paguristes tortugae* explains the asymmetry and gaps in the anomuran histograms. A review of crustacean barcodes found that the GC% varies significantly amongst families (Matzen da Silva et al. 2011), ranging from ~30-50% with family-specific averages ranging from 33-42% (Matzen da Silva et al. 2011). If AT/GC bias is similar across the mitochondrion, one might expect that the GC% of COI and 16S are correlated and deviations from the trend-line could be another way to identify potential pseudogenes. Our data show an overall positive correlation between the two, considerable scatter around the trend-line and a small cluster of carideans that fall somewhat below the other carideans, but still within the overall variation for the decapods (Fig. 5).

Our limited ability to identify pseudogene sequences without cloning indicates that pseudogenes are likely to infiltrate metabarcoding datasets generated by high-throughput sequencing, as well as datasets generated by sanger sequencing. One concern about the inclusion of pseudogenes in these kinds of biodiversity studies is that they may over-estimate the number of OTUs reported (Schneider-Broussard and Neigel 1997, Williams and Knowlton 2001, Song et al. 2008, Schubart 2010, Matzen da Silva et al. 2011, Raupach and Radulovici 2015). This could certainly happen, but a more common occurrence is that either co-amplification of the gene and its pseudogenes reduces the quality of the reads, resulting in an unusable sequence or a single sequence significantly out-amplifies the other, resulting in a single sequence from each species. If this is the pseudogene, the results could complicate phylogenetic analyses, but are unlikely to impact the results of DNA barcoding studies. One situation where pseudogenes certainly will not impact the efficacy of a DNA barcoding approach is in the identification of unknown samples through comparisons with sequences from carefully identified material. If a sequence is known to come from a specific species, whether or not it is a pseudogene, that sequence can be used to generate a positive identification of unknown material. Therefore, rather than discard potential pseudogene sequences, they should be included in barcode databases as a potentially informative resource (see also the arguments by Schizas 2012).

### Taxonomy Training and DNA Barcoding

The present study, along with Cancian de Araujo et al. (2018), demonstrates that DNA barcoding of common species encountered during field training in tropical biodiversity can contribute useful data to the effort to barcode metazoans. Such data, despite collected from a single site, may be relevant throughout the Caribbean as connectivity is considered high within this sea for some decapods [e.g. *Panulirus argus* (Naro-Maciel et al. 2011, Kough et al. 2013)]. With only a moderate collecting effort (i.e. incidental collections over 4 weeks total), we obtained a barcode dataset of a similar number of decapod OTUs as the exhaustive decapod DNA barcode dataset for the North Sea (Raupach et al. 2015). Of these, 32% of the COI sequences and 24% of the 16S sequences were new to GenBank and 39% of the BINs were new to BOLD. It has previously been noted that crustacean sequences are poorly represented in BOLD (Raupach and Radulovici 2015). The number of new OTUs is perhaps significantly lower than what would be expected in locations that have previously received less intensive systematic study than Bocas del Toro, which has been the focus of alpheid shrimp systematics for over 15 years (Williams and Knowlton 2001, Anker et al. 2007, Anker et al. 2008, Mathews and Anker 2009, Anker 2010, Anker et al. 2012). Nevertheless, a significant portion of the sequenced species generated new species records in GenBank and required little additional effort in the field over and above the collection and identification exercises already underway. Of course, as with any vouchered material, additional curatorial effort was necessary compared to typical field courses where the material is not usually vouchered.

## Figures and Tables

**Figure 3a.**
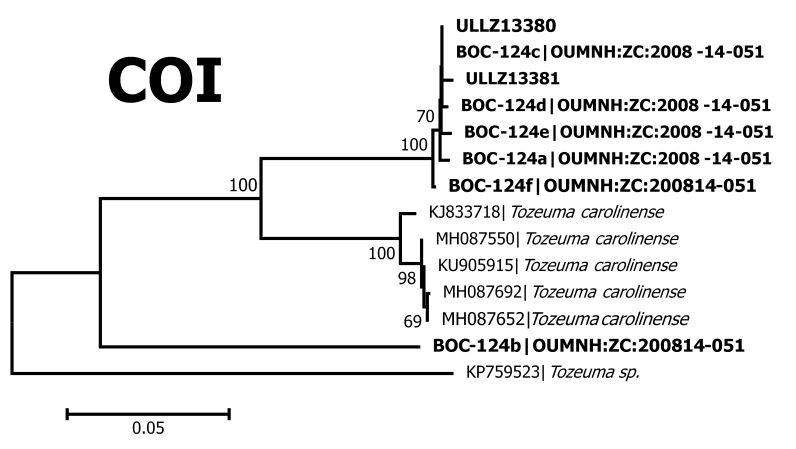
Neighbour-Joining tree for *cytochrome c oxidase* subunit I

**Figure 3b.**
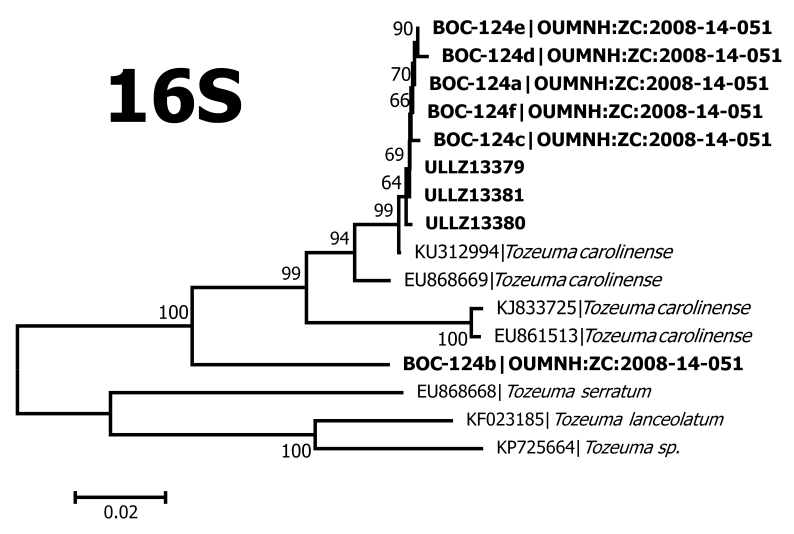
Neighbour-Joining tree for 16S ribosomal RNA

**Table 1.** Summary of the Operational Taxonomic Units (OTUs) detected in this study, their morphological identification, Barcode Index Numbers (BINs) in BOLD and their contribution to information in GenBank. Whenever our OTU provided a new species name in GenBank, the morphospecies was tagged with the symbols ^ or ~ if the contribution occurred in COI or 16S, respectively. New BINs generated in this study are indicated with asterisks*.

**Family**	**Morphospecies**	**Museum ID**	**BINs**	#**Inds**	**COI new to GenBank (<95% identity)**	**16S new to GenBank (<97% identity)**
**Porcellanid Crabs: Anomura**						
Albuneidae	Lepidopa cf. richmondi	ULLZ13327	BOLD:ACT9781*	1	NEW	NEW
Diogenidae	*Calcinus tibicen*	ULLZ13427, ULLZ13444, ULLZ13426, ULLZ13445	BOLD:AAE8392	4	-	-
Diogenidae	*Clibanarius antillensis*	ULLZ13597, ULLZ13599, ULLZ13619, ULLZ13617, ULLZ13618	BOLD:AAK1039	5	-	-
Diogenidae	*Clibanarius sclopetarius*	ULLZ13353	BOLD:ACB6405	1	-	-
Diogenidae	*Dardanus fucosus*^	ULLZ13352, ULLZ13602	BOLD:AAI5823	2	NEW	NEW
Diogenidae	*Paguristes tortugae*^	ULLZ13663, ULLZ13664, ULLZ13665, ULLZ13707, ULLZ13708, ULLZ13709	BOLD:ACT9547*	6	-	NEW
Hippidae	*Emerita* sp.	ULLZ13325, ULLZ13456, ULLZ13457, ULLZ13690	BOLD:ACU0009	4	-	-
Hippidae	*Hippa testudinaria*^~	ULLZ13326, ULLZ13706, ULLZ13613	BOLD:ACT9780*	3	NEW	NEW
Paguridae	*Pagurus brevidactylus*	ULLZ13660	BOLD:AAF9919	1	NEW	NEW
Paguridae	*Pagurus criniticornis*	ULLZ13635	BOLD:ACU0173*	1	NEW	NEW
Paguridae	*Pagurus criniticornis*, *P. nr. criniticornis*	ULLZ13482, ULLZ13483a, ULLZ13483b, ULLZ13633, ULLZ13634, ULLZ13692, ULLZ13693, ULLZ13714, ULLZ13632	BOLD:ACT9783*	9	NEW	NEW
Paguridae	*Pagurus nr. maclaughlinae*^	ULLZ13384, ULLZ13385	BOLD:ACT9865	2	NEW	NEW
Porcellanidae	*Megalobrachium roseum*	ULLZ13449, ULLZ13450	BOLD:ACT9407	2	-	-
Porcellanidae	*Pachycheles chacei*^	ULLZ13436, ULLZ13437, ULLZ13438	BOLD:ACT9930*	3	NEW	-
Porcellanidae	*Pachycheles cristobalensis*^~	ULLZ13421, ULLZ13428, ULLZ13429, ULLZ13430	BOLD:ACU0707*	4	NEW	-
Porcellanidae	*Pachycheles tuerkayi*^	ULLZ13354, ULLZ13586, ULLZ13587, ULLZ13593, ULLZ13595, ULLZ13701	BOLD:ACU0397*	6	NEW	-
Porcellanidae	*Petrolisthes armatus*	ULLZ13315, ULLZ13369, ULLZ13370, ULLZ13371, ULLZ13433, ULLZ13434	BOLD:AAA2699	6	-	-
Porcellanidae	*Petrolisthes galathinus*	ULLZ13339, ULLZ13454, ULLZ13612, ULLZ13672, ULLZ13673, ULLZ13674	BOLD:ACG7994	6	-	-
Porcellanidae	*Petrolisthes jugosus*	ULLZ13423, ULLZ13424	BOLD:ACU0611*	2	NEW	NEW
**Brachyuran Crabs: Brachyura**						
Calappidae	*Calappa galloides*~	ULLZ13575	BOLD:AAV0354	1	-	-
Calappidae	*Calappa ocellata*	ULLZ13404	BOLD:ACT9710	1	-	-
Epialtidae	*Acanthonyx petiverii*	ULLZ13398, ULLZ13399, ULLZ13422	BOLD:ACG7625	3	-	-
Epialtidae	*Epialtus bituberculatus*	ULLZ13572, ULLZ13573, ULLZ13574, ULLZ13603, ULLZ13601, ULLZ13604	BOLD:ACG7953	6	-	-
Epialtidae	*Macrocoeloma diplacanthum*^~	ULLZ13313	BOLD:ACT8809*	1	NEW	NEW
Epialtidae	*Macrocoeloma subparellelum*^~	ULLZ13311	BOLD:ACT9697*	1	NEW	NEW
Epialtidae	*Macrocoeloma trispinosum*	ULLZ13374, ULLZ13375, ULLZ13455, ULLZ13667	BOLD:ACG7680	4	-	-
Epialtidae	*Pitho mirabilis*	ULLZ13592	BOLD:ACT8761*	1	NEW	NEW
Eriphiidae	*Eriphia gonagra*	ULLZ13395, ULLZ13396, ULLZ13397	BOLD:ACG8098	3	-	-
Gecarcinidae	*Cardisoma guanhumi*	ULLZ13341	BOLD:ACT8737	1	-	-
Grapsidae	*Goniopsis cruentata*	ULLZ13661	BOLD:ACG7928	1	-	-
Grapsidae	*Grapsus grapsus*^	ULLZ13414, ULLZ13415, ULLZ13416	BOLD:ACU0001*	3	-	-
Grapsidae	*Pachygrapsus gracilis*	ULLZ13358, ULLZ13656	BOLD:ACU0366	2	-	-
Grapsidae	*Pachygrapsus transversus*	ULLZ13606, ULLZ13607, ULLZ13608	BOLD:AAG9839	3	-	-
Inachidae	*Stenorhynchus seticornis*	ULLZ13359, ULLZ13360, ULLZ13400	BOLD:AAJ5290	3	-	-
Inachoididae	*Inachoides* sp.	ULLZ13463	BOLD:ACT9182*	1	NEW	-
Leucosiidae	*Speloeophorus nodosus*^	ULLZ13317	BOLD:ACT8927*	1	NEW	-
Majidae	*Thoe puella*	ULLZ13582, ULLZ13583	BOLD:ACU0496*	2	-	-
Menippidae	*Menippe nodifrons*	ULLZ13481, ULLZ13654, ULLZ13655	BOLD:AAX4629	3	-	-
Mithracidae	*Amphithrax aculeatus*	ULLZ13596	BOLD:ACU0682	1	-	-
Mithracidae	*Mithraculus cinctimanus*	ULLZ13328, ULLZ13408, ULLZ13464, ULLZ13467	BOLD:ACG7379	4	-	-
Mithracidae	*Mithraculus coryphe*	ULLZ13318, ULLZ13460, ULLZ13461, ULLZ13621	BOLD:ACT9266*	4	-	-
Mithracidae	*Mithraculus forceps*	ULLZ13361, ULLZ13363, ULLZ13571, ULLZ13675, ULLZ13568, ULLZ13569	BOLD:AAC9888	6	-	-
Mithracidae	*Mithrax hispidus*	ULLZ13391, ULLZ13453, ULLZ13682	BOLD:ACU0360*	3	-	-
Mithracidae	*Mithrax pleuracanthus*	ULLZ13348, ULLZ13362, ULLZ13435, ULLZ13705	BOLD:ACB5456	4	-	NEW
Mithracidae	*Omalacantha bicornuta*	ULLZ13377, ULLZ13378, ULLZ13562, ULLZ13563, ULLZ13564, ULLZ13567, ULLZ13662, ULLZ13376, ULLZ13565, ULLZ13566	BOLD:AAX4083	10	-	-
Ocypodidae	*Minuca burgersi*	ULLZ13367	BOLD:ACG7755	1	-	-
Ocypodidae	*Minuca rapax*	ULLZ13366, ULLZ13368, ULLZ13441, ULLZ13442	BOLD:ACT8667	4	-	-
Ocypodidae	*Ocypode quadrata*	ULLZ13411	BOLD:ACU0659	1	-	-
Panopeidae	*Acantholobulus bermudensis*	ULLZ13329a, ULLZ13329b	BOLD:ACG8166	2	-	-
Panopeidae	*Eurypanopeus abbreviatus*	ULLZ13590	BOLD:ACU0495	1	-	-
Panopeidae	*Eurytium limosum*	ULLZ13382, ULLZ13383, ULLZ13473	BOLD:ACT8759	3	-	-
Panopeidae	*Panopeus lacustris*	ULLZ13686	BOLD:ACU0442	1	-	-
Panopeidae	*Panopeus occidentalis*	ULLZ13344, ULLZ13694	BOLD:AAX2632	2	-	-
Percnidae	*Percnon gibbesi*	ULLZ13443, ULLZ13479	BOLD:AAC3992	2	-	-
Pilumnidae	*Pilumnus caribaeus*	ULLZ13393, ULLZ13394	BOLD:ACG8072	2	-	-
Pilumnidae	*Pilumnus dasypodus*, *P. caribaeus*	ULLZ13440, ULLZ13616, ULLZ13439, ULLZ13614, ULLZ13615	BOLD:AAI2968	5	-	-
Pilumnidae	*Pilumnus gemmatus*	ULLZ13462, ULLZ13680, ULLZ13681	BOLD:AAY4016	3	-	-
Pilumnidae	*Pilumnus holosericus*	ULLZ13577, ULLZ13578	BOLD:ACU1343	2	-	-
Pilumnidae	*Pilumnus nudimanus*	ULLZ13466	BOLD:ACT9365	1	-	-
Pilumnidae	*Pilumnus pannosus*, *P. reticulatus*	ULLZ13581, ULLZ13589		2	-	-
Pinnotheridae	*Austinixa aidae*	ULLZ13459	BOLD:ACU0215*	1	NEW	-
Pinnotheridae	*Austinixa* sp.	ULLZ13644, ULLZ13642, ULLZ13643, ULLZ13332	BOLD:ACU0214*, BOLD:ACU0213*	4	NEW	-
Pinnotheridae	*Tunicotheres moseri*^	ULLZ13678	BOLD:ACT9535*	1	NEW	-
Plagusiidae	*Plagusia depressa*^	ULLZ13403	BOLD:ACT9499*	1	NEW	-
Portunidae	*Achelous sebae*^	ULLZ13477	BOLD:ACG7575	1	NEW	-
Portunidae	*Callinectes danae*	ULLZ13321, ULLZ13373, ULLZ13409, ULLZ13410, ULLZ13700	BOLD:ACD2797	5	-	-
Portunidae	*Callinectes larvatus*	ULLZ13372, ULLZ13392, ULLZ13405, ULLZ13406, ULLZ13407, ULLZ13476	BOLD:ACC4630	6	-	-
Portunidae	*Charybdis hellerii*	ULLZ13355, ULLZ13465, ULLZ13584, ULLZ13585	BOLD:AAO9264	4	-	-
Pseudothelphusidae	*Ptychophallus* sp.	ULLZ13471, ULLZ13684	BOLD:ACU0372	2	-	-
Sesarmidae	*Aratus pisonii*	ULLZ13365, ULLZ13364	BOLD:ACG8032	2	-	-
Sesarmidae	*Armases ricordi*	ULLZ13452, ULLZ13579, ULLZ13580	BOLD:ACT9799*	3	NEW	-
Sesarmidae	*Sesarma curacaoense*	ULLZ13294, ULLZ13388, ULLZ13389, ULLZ13390	BOLD:ACT9653	4	-	-
Varunidae	*Cyclograpsus integer*	ULLZ13431, ULLZ13691	BOLD:ACT8869	2	-	-
Xanthidae	*Cataleptodius floridanus*	ULLZ13349, ULLZ13417, ULLZ13418, ULLZ13419	BOLD:AAI1248	4	-	-
Xanthidae	*Paraliomera dispar*	ULLZ13413	BOLD:ACH4832	1	-	-
**Shrimps: Caridea, Dendrobranchiata and Stenopodidea**						
Alpheidae	*Alpheus* sp.		BOLD:AAH8594	1	-	-
Alpheidae	*Alpheus armillatus*	OUMNH:ZC:2008-14-117	BOLD:ADP1810*	1	NEW	-
Alpheidae	*Alpheus angulosus*	OUMNH:ZC:2008-14-082, OUMNH:ZC:2008-14-095, OUMNH:ZC:2008-14-100, ULLZ13652	BOLD:AAC6145*	11	-	-
Alpheidae	*Alpheus bahamensis*~	OUMNH:ZC:2008-14-079, OUMNH:ZC:2008-14-080, ULLZ13645, ULLZ13646, ULLZ13647	BOLD:AAC6728*	9	NEW	NEW
Alpheidae	*Alpheus cristulifrons*~	OUMNH:ZC:2008-14-089, OUMNH:ZC:2009-14-084	BOLD:ADP1409*	3	-	NEW
Alpheidae	*Alpheus estuariensis*	OUMNH:ZC:2008-14-083	BOLD:ADC6108	1	-	-
Alpheidae	*Alpheus floridanus*	ULLZ13478	BOLD:ACU1957*	1	NEW	-
Alpheidae	*Alpheus nuttingi*	ULLZ13314A, ULLZ13314B, ULLZ13622, ULLZ13625, ULLZ13649, ULLZ13650, ULLZ13624	BOLD:ACU0031*	7	-	-
Alpheidae	*Alpheus packardii complex*^	OUMNH:ZC:2008-14-081, OUMNH:ZC:2008-14-108, OUMNH:ZC:2008-14-111	BOLD:ACQ5750	6	-	NEW
Alpheidae	*Alpheus packardii complex*^	OUMNH:ZC:2008-14-081	BOLD:AAC6138	1	NEW	NEW
Alpheidae	*Alpheus packardii complex* sp. 1^	OUMNH:ZC:2008-14-084, OUMNH:ZC:2008-14-085, ULLZ13447, ULLZ13626, ULLZ13628, ULLZ13630, ULLZ13631, OUMNH:ZC:2008-14-081	BOLD:AAH7067*	9	NEW	NEW
Alpheidae	*Alpheus packardii complex* sp. 2^	ULLZ13627	BOLD:ACT9784*	1	NEW	NEW
Alpheidae	*Alpheus paracrinitus*~	OUMNH:ZC:2008-14-118, OUMNH:ZC:2018-14-105, OUMNH:ZC:2018-14-107	BOLD:ADP3337*	4	NEW	NEW
Alpheidae	*Alpheus paracrinitus*~	OUMNH:ZC:2008-14-118	BOLD:ADP0639*	1	NEW	NEW
Alpheidae	*Alpheus paraformosus*~	OUMNH:ZC:2008-14-087	BOLD:AAC6141	1	-	-
Alpheidae	*Alpheus peasei*~	OUMNH:ZC:2008-14-086, OUMNH:ZC:2008-14-090, OUMNH:ZC:2008-14-114:	BOLD:ADP2822*	3	-	NEW
Alpheidae	*Alpheus thomasi*	ULLZ13320, ULLZ13629	BOLD:ACT9880	2	-	-
Alpheidae	*Alpheus viridari*	OUMNH:ZC:2008-14-094, OUMNH:ZC:2008-14-110, ULLZ13677	BOLD:AAI2078	3	-	-
Alpheidae	Automate aff. dolichognatha^	OUMNH:ZC:2008-14-109	BOLD:ADO9733*	1	NEW	NEW
Alpheidae	*Synalpheus brevicarpus*	ULLZ13639, ULLZ13640	BOLD:ACC9307	2	-	-
Alpheidae	*Synalpheus fritzmuelleri*	OUMNH:ZC:2008-14-127	BOLD:ADP1424*, BOLD:AAG9019	3	NEW	NEW
Alpheidae	*Synalpheus hemphilli*	ULLZ13641	BOLD:ADP2538*	3	NEW	NEW
Alpheidae	*Synalpheus yano*	OUMNH:ZC:2008-14-133	BOLD:AAC6139	24	-	-
Alpheidae	*Synalpheus nr. yano*		BOLD:ADP3823*	4	NEW	NEW
Alpheidae	*Synalpheus nr. yano*	OUMNH:ZC:2008-14-133	BOLD:ACC9017	2	-	NEW
Alpheidae	*Synalpheus nr. yano*		BOLD:ACC9109	2	-	-
Alpheidae	*Synalpheus nr. yano*	OUMNH:ZC:2008-14-103	BOLD:AAC6142	1	-	-
Alpheidae	*Synalpheus nr. yano*		BOLD:ADP1425	1	-	-
Alpheidae	*Synalpheus nr. yano*	OUMNH:ZC:2008-14-133	BOLD:ADP4347*	6	-	-
Alpheidae	*Synalpheus nr. yano*	OUMNH:ZC:2008-14-133	BOLD:ADP4348*	2	-	-
Alpheidae	*Synalpheus dardeaui*	OUMNH:ZC:2008-14-133, ULLZ13488	BOLD:AAE5682	10	-	-
Alpheidae	*Synalpheus ul*, *S. longicarpus*	OUMNH:ZC:2008-14-127, ULLZ13710	BOLD:AAG9018	4	-	-
Alpheidae	*Synalpheus apioceros*	OUMNH:ZC:2008-14-091	BOLD:ADO8257*, BOLD:AAD6588	5	-	NEW
Alpheidae	*Synalpheus guerini*		BOLD:AAG9015	3	-	-
Alpheidae	*Synalpheus scaphoceris*		BOLD:AAF9341	4	-	-
Atyidae	*Potimirim glabra*	OUMNH:ZC:2008-14-014	BOLD:ACI0486	1	-	-
Hippolytidae	*Hippolyte obliquimanus*	OUMNH:ZC:2008-14-043, OUMNH:ZC:2008-14-045, ULLZ13696, ULLZ13697	BOLD:AAE4017	7	-	-
Hippolytidae	*Tozeuma carolinense*	OUMNH:ZC:2008-14-051, ULLZ13380, ULLZ13381, ULLZ13379	BOLD:ACU0079*	8	NEW	-
Hippolytidae	*Tozeuma carolinense*	OUMNH:ZC:2008-14-051	BOLD:ADP3533*	1	NEW	NEW
Palaemonidae	*Brachycarpus biunguiculatus*	OUMNH:ZC:2008-14-038, OUMNH:ZC:2008-14-068	BOLD:AAE0296	2	NEW	-
Palaemonidae	*Cuapetes americanus*	ULLZ13670, OUMNH:ZC:2008-14-032, ULLZ13668, ULLZ13671, ULLZ13712, ULLZ13715, ULLZ13669, ULLZ13713	BOLD:ACG8330, BOLD:AAI2206	10	-	-
Palaemonidae	*Leander paulensis*	ULLZ13685		1	-	-
Palaemonidae	*Leander tenuicornis*	OUMNH:ZC:2008-14-034, OUMNH:ZC:2008-14-036, OUMNH:ZC:2008-14-067, ULLZ13351, ULLZ13485a, ULLZ13485b, ULLZ13486a, ULLZ13486b	BOLD:AAC8465	10	NEW	-
Palaemonidae	*Palaemon northropi*	OUMNH:ZC:2008-14-031, ULLZ13356	BOLD:AAG9010	2	-	-
Palaemonidae	*Periclimenaeus schmitti*^~	OUMNH:ZC:2008-14-064, OUMNH:ZC:2008-14-072, OUMNH:ZC:2008-14-073, OUMNH:ZC:2008-14-074, OUMNH:ZC:2008-14-075, OUMNH:ZC:2008-14-076, OUMNH:ZC:2008-14-139	BOLD:ADP1635*	8	NEW	NEW
Palaemonidae	*Periclimenes rathbunae*	ULLZ13699	BOLD:AAC6144	1	-	-
Palaemonidae	*Periclimenes yucatanicus*	ULLZ13472, ULLZ13345, ULLZ13704, ULLZ13346, ULLZ13695	BOLD:ADC8100*, BOLD:ADC8099*, BOLD:ACU2547*, BOLD:AAH8593*	5	-	-
Palaemonidae	*Typton carneus*^~	ULLZ13711	BOLD:ACU1120*	1	NEW	NEW
Palaemonidae	Typton cf. distinctus^~	ULLZ13448, ULLZ13698	BOLD:ACT9785*	2	NEW	NEW
Penaeidae	*Metapenaeopsis gerardoi*~	ULLZ13357	BOLD:ACT9874	1	NEW	NEW
Processidae	*Processa bermudensis*^	OUMNH:ZC:2008-14-059, ULLZ13333	BOLD:AAJ2144*	2	NEW	-
Processidae	*Processa fimbriata*^	OUMNH:ZC:2008-14-056	BOLD:AAF3128	2	NEW	NEW
Sicyoniidae	*Sicyonia* sp.		BOLD:ADO8841*	2	NEW	NEW
Sicyoniidae	*Sicyonia* sp.		BOLD:ADO8840*	1	NEW	-
Sicyoniidae	*Sicyonia laevigata*	ULLZ13702	BOLD:ACT9954	1	-	-
Sicyoniidae	*Sicyonia laevigata*	ULLZ13484, ULLZ13703	BOLD:AAF9340	4	-	-
Spongicolidae	*Microprosthema semilaeve*^	OUMNH:ZC:2008-14-052, OUMNH:ZC:2008-14-053, ULLZ13648, ULLZ13716	BOLD:AAD8095*	4	NEW	-
Stenopodidae	*Stenopus hispidus*	ULLZ13666, OUMNH:ZC:2008-14-076, ULLZ13676	BOLD:AAC8463	3	-	-
Stenopodidae	*Stenopus scutellatus*	OUMNH:ZC:2008-14-047	BOLD:ADD4717	1	-	-
**Mudshrimp: Axiidea**						
Callianassidae	*Neocallichirus grandimana*^	NHMW 25285, NHMW 25286	BOLD:AAG5150*	2	NEW	-
Callianassidae	*Neocallichirus guassutinga*^	NHMW 25287, ULLZ13322, ULLZ13323, ULLZ13324	BOLD:AAN0153	4	-	-
Callianassidae	*Neocallichirus maryae*^	ULLZ13474	BOLD:ACT9837*	1	NEW	-
Callianassidae	*Pseudobiffarius caesari*^~	ULLZ13480	BOLD:ACU0753*	1	NEW	NEW
**Mudshrimp: Gebiidea**						
Upogebiidae	*Upogebia corallifora*^	ULLZ13683	BOLD:ACT9886*	1	NEW	NEW
**Lobsters: Achelata**						
Palinuridae	*Panulirus argus*	ULLZ13319, ULLZ13458	BOLD:ACD2165, BOLD:AAL9182	2	-	-
